# Complement System Inhibitors in Nephrology: An Update—Narrative Review

**DOI:** 10.3390/ijms26125902

**Published:** 2025-06-19

**Authors:** Mugurel Apetrii, Alexandru Dan Costache, Irina Iuliana Costache Enache, Luminita Voroneanu, Andreea Simona Covic, Mehmet Kanbay, Adrian Covic

**Affiliations:** 1 Faculty of Medicine, “Grigore T. Popa” University of Medicine and Pharmacy, 700115 Iasi, Romania; mugurel.apetrii@gmail.com (M.A.); ii.costache@yahoo.com (I.I.C.E.); lumivoro@yahoo.com (L.V.); andreea.covic@gmail.com (A.S.C.); accovic@gmail.com (A.C.); 2 “Dr. C.I. Parhon” Clinical Hospital, 700503 Iasi, Romania; 3 Clinical Rehabilitation Hospital, 700661 Iasi, Romania; 4 “St. Spiridon” Emergency County Hospital, 700111 Iasi, Romania; 5 School of Medicine, Koç University, Istanbul 34450, Turkey; drkanbay@yahoo.com

**Keywords:** complement system, C3 glomerulopathy, immune complex membranoproliferative glomerulonephritis, atypical hemolytic uremic syndrome

## Abstract

Complement system inhibitors are emerging as promising therapies in nephrology, particularly for diseases where complement dysregulation is central to pathogenesis. This review summarizes the role of complement activation in kidney diseases and current evidence supporting complement-targeted treatments. As the complement system can be involved in the pathogenesis of different diseases to varying degrees, several research works have been conducted. These research efforts aim, firstly, to understand the mechanisms and role of complement cascade components in the most prevalent nephrological diseases and, secondly, to explore the potential of complement system inhibitors in these conditions and their possible clinical applications. Clinical trials demonstrate that complement inhibitors are most effective in conditions with significant complement involvement, such as C3 glomerulopathy (C3G), atypical hemolytic uremic syndrome (aHUS), and immune complex membranoproliferative glomerulonephritis (IC-MPGN). These agents show variable benefits in diseases with partial complement activation, including lupus nephritis and ANCA-associated vasculitis, while their role in disorders like diabetic nephropathy and focal–segmental glomerulosclerosis remains limited. Complement inhibition offers a targeted strategy to prevent disease progression and improve outcomes in selected nephrological disorders.

## 1. Introduction

### The Basics of Complement System

The complement system, a critical component of innate immunity, plays a central role in host defense and inflammatory responses. Its dysregulation is increasingly recognized as a driver of several glomerular diseases. Understanding complement pathways—classical, lectin, and alternative—is essential to appreciate their contribution to kidney pathology and the therapeutic rationale for complement inhibition [1,2,3].

**Figure 1:**
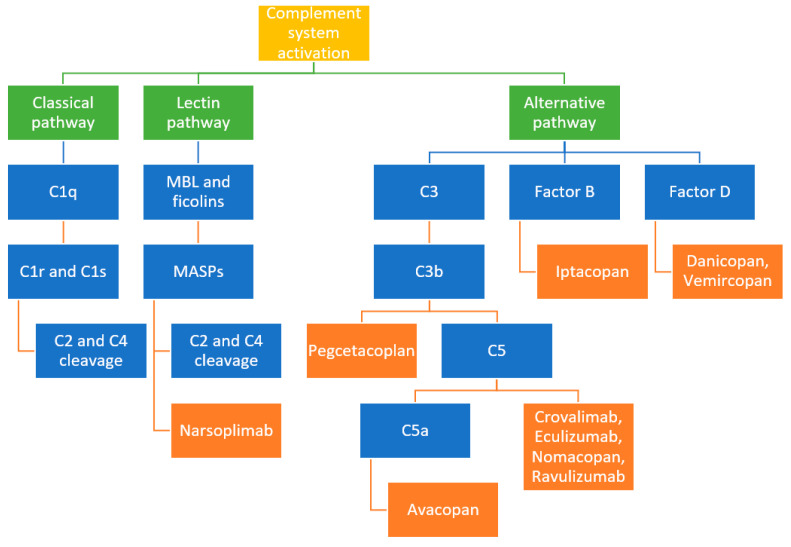
The complement system activation pathways and the first components involved.

All three pathways lead to the formation of the two main enzymes in the cascade, the C3 and C5 convertases, which in turn contribute to the generation of anaphylatoxins C3a and C5a, the terminal complement complex (TCC) or the membrane attack complex (MAC), and various opsonins (e.g., C4b, C4d, C3b, iC3b, C3dg, and C3d) [1,4] (see Figure 1).

In order for the activation of the complement components to occur, they require a stimulus such as pathogen-associated molecular patterns (PAMPs), i.e., an infectious agent (e.g., bacteria) or damage-associated molecular patterns (DAMPs), secondary to cellular or tissue damage [1].

Complement is continuously active at low levels through processes such as the alternative pathway “tick-over” and classical pathway engagement by natural IgM antibodies, allowing the immune system to monitor for pathogens. However, this constant activation necessitates precise regulation to avoid damage to host tissues. These regulators act in a manner analogous to anticoagulant proteins (e.g., proteins C and S) that modulate the coagulation cascade to prevent unwarranted thrombin generation [1] (see Table 1).

Failures in complement regulation due to genetic mutations or autoantibodies can lead to uncontrolled activation, resulting in tissue injury. This mechanism underlies complement-mediated thrombotic microangiopathies (CM-TMA), including atypical hemolytic uremic syndrome (aHUS). Genetic variants affecting complement regulation are more prevalent than autoantibodies in adults with CM-TMA [1].

Pathogenic sequence variants have been identified in several key complement genes, including CFH, CFI, MCP (CD46), C3, and complement factor B (CFB). These variants disrupt normal regulation of the alternative pathway, predisposing individuals to complement overactivation and subsequent endothelial damage. These genetic abnormalities contribute to the development of TMA and aHUS, and understanding them has been critical for developing targeted complement inhibitors [1,2].

Hereditary CM-TMA due to complement gene variants can present across all age groups. In the French national cohort, Frémeaux-Bacchi et al. identified complement gene mutations in approximately 60% of 214 patients, with CFH, CFI, and MCP being the most frequently involved genes. Variants in CFI and CFH were more commonly observed in adult-onset cases, whereas MCP mutations tended to present earlier in life. Although the sample size limited definitive genotype–phenotype conclusions, the study revealed that adults were more likely to present with severe renal involvement and experienced significantly higher rates of progression to end-stage kidney disease (ESKD)—46% in adults versus 16% in children at one year—despite similar mortality rates across age groups [4].

Complementing these findings, the KDIGO Controversies Conference reports—particularly those by Goodship et al. (2017) and Vivarelli et al. (2024)—emphasized the critical importance of comprehensive complement genetic testing and early recognition of high-risk genotypes, especially in cases lacking an apparent secondary trigger. These reports underscore the role of complement dysregulation in driving both initial disease onset and recurrence after kidney transplantation, reinforcing the utility of complement inhibitors in selected genetic profiles [5,6]. Additionally, data from Johns Hopkins University support these observations, showing a median age at presentation of approximately 46 years in adult patients with TMA and AKI and a female predominance [1,3]. Collectively, these datasets provide converging evidence that genetic variants in complement regulatory pathways are central to disease progression and clinical decision-making in CM-TMA.

Collectively, these findings underscore the importance of recognizing complement dysregulation as a central factor in TMAs and other complement-mediated renal diseases. Genetic screening for complement gene variants is essential in patients presenting with TMA, especially when secondary causes are absent or unclear. Furthermore, these insights provide a strong rationale for the use of complement inhibitors, particularly C5 blockers like eculizumab and Ravulizumab, in managing CM-TMA and preventing progression to ESKD [1,7].

## 2. The Involvement of the Complement System in Nephrological Diseases

Complement-mediated kidney injury occurs via immune complex deposition, generation of anaphylatoxins, and formation of membrane attack complexes (MAC). The kidney’s capacity for local complement synthesis and regulation makes it vulnerable to complement-mediated damage. With the advent of targeted inhibitors, modulating complement activation has become a viable therapeutic strategy for diseases characterized by excessive or uncontrolled complement activity [7,8].

Apart from the systemic activation of the complement system, during inflammatory responses or ischemia–reperfusion injury (IRI), several of its proteins can be upregulated locally in renal cells. For example, during inflammatory processes, an increase in C3 from 5% to 16% has been observed in all renal cell lines [7,8].

However, the degree of involvement of the complement in the pathogenesis of certain nephrological diseases varies and with it also the potential of complement-inhibition therapies in treating them. Several diseases have a significant complement system involvement in their pathogenesis and, therefore, respond better and show greater remission under complement inhibitors treatment, such as C3-glomerulopathy (C3G), atypical hemolytic–uremic syndrome (aHUS), systemic lupus erythematosus (SLE), and the primary immune complex membranoproliferative glomerulonephritis (IC-MPGN). Less involvement of the complement and, therefore, less impact of the therapeutic inhibitors have been shown by the anti-neutrophil cytoplasmic antibody (ANCA)-associated vasculitis (AAV), immunoglobulin A (IgA) nephropathy (IgAN), IgA-associated vasculitis with nephritis (IgAVN), antiphospholipid antibody syndrome (AAS), membranous nephropathy (MN), secondary thrombotic microangiopathy (TMA), and secondary membranoproliferative glomerulonephritis (MPGN). Finally, diabetic nephropathy (DN) and focal–segmental glomerulosclerosis (FSGS) exhibit little involvement of complement components. Also, in these situations, complement inhibition therapy has a more protective role, rather than curative [5,6,9] (see Table 2).

### 2.1. Atypical Hemolytic Uremic Syndrome (aHUS)

aHUS involves uncontrolled alternative pathway activation, leading to thrombotic microangiopathy with hemolytic anemia, thrombocytopenia, and acute kidney injury. Clinically, aHUS has a prodrome consisting of fever, diarrhea, and respiratory tract symptoms, considering its onset is secondary to digestive or respiratory tract infections. Patients have hypertension, hemolytic anemia, thrombocytopenia, and acute kidney injury, while extra-renal manifestations involve the respiratory tract (e.g., pulmonary hemorrhage), the digestive tract (e.g., diarrhea), the central nervous system (e.g., stroke), the heart (e.g., myocarditis), the skin (e.g., purpura), and the eyes (retinal hemorrhage). Neurologic manifestations such as seizures or altered consciousness, gastrointestinal involvement like pancreatitis or bleeding, and cardiac complications, including cardiomyopathy, may also occur [9].

Most forms of aHUS are driven by dysregulations in the alternative and terminal pathways, such as infections, pregnancy, autoimmune conditions, drugs (e.g., calcineurin inhibitors, chemotherapy), interferon β administration, vascular endothelial growth factor inhibition, and diacylglycerol kinase-ε and cobalamin-C deficiencies, among other factors. Inborn errors of metabolism, especially involving cobalamin C, are more common in infants and should be considered in young children presenting with HUS [10,11].

The pathogenesis involves an imbalance in genes responsible for complement regulatory proteins, either through gain of function (e.g., C3) or loss of function (e.g., CFH). Mutations of these genes are found in most patients with aHUS, along with anti-CFH antibodies or defects in genes encoding CFH-related proteins. The most commonly affected genes include CFH (20–30%), CD46 (5–15%), CFI (4–10%), C3 (2–10%), and CFB (1–4%). Thrombomodulin gene (THBD) variants and deletions in CFHR genes (e.g., CFHR1, CFHR3) have also been identified [9].

The diagnosis is best established through molecular analysis, which also helps in guiding treatment. Low C3 with normal C4 suggests alternative pathway dysregulation, while low CH50 and factor B levels may further support complement involvement. Worst clinical outcomes and higher recurrence rates are encountered in patients with CFH mutations. However, given that such mutations are not found in all patients and genetic testing is limited, diagnosis should also involve clinical and laboratory evaluations. Additionally, family history, age of onset (<1 year or during pregnancy), and prior episodes may guide suspicion toward aHUS [9].

The available targeted treatment options in aHUS are plasma therapy and complement blockade with an anti-C5 monoclonal antibody. Eculizumab remains the mainstay therapy in most patients, showing favorable outcomes in preventing recurrence and preserving renal function. Also, patients require supportive care, i.e., hypertension control, correction of anemia and thrombocytopenia, as well as the treatment of complications, including renal replacement therapy. In patients with anti-CFH antibodies, plasma exchange followed by immunosuppressive therapy (e.g., corticosteroids, rituximab, or mycophenolate mofetil) is considered effective, particularly in preventing relapse [9].

### 2.2. C3-Glomerulopathy

C3G is a pathological category that defines a group of rare kidney diseases characterized by complement dysregulation in the glomerular microenvironment, often accompanied by measurable complement dysregulation in the circulation. The disease is typically driven by immunological and/or genetic factors, although a significant number of patients have no identifiable disease drivers with current testing methods [12].

C3 glomerulopathy is probably the most representative kidney disease involving the complement system. Histologically, it is characterized by a membranoproliferative pattern. On light microscopy, several patterns can be observed, such as sclerosing and crescentic, mesangial, and endocapillary proliferative patterns [6].

On immunofluorescence microscopy, the main characteristic is the dominance of C3 with negative C1q. C3 predominance of at least two orders of magnitude over other immunoreactants is considered diagnostic. On electron microscopy, dense deposit disease (DDD) shows pathognomonic intramembranous electron-dense material within the glomerular basement membrane (GBM), whereas C3 glomerulonephritis (C3GN) reveals subendothelial and mesangial deposits of lesser density [6].

In the renal biopsies of C3G patients, type 1 factor H-related (FHR) proteins, particularly FHR1 and FHR5, are frequently observed. Additionally, pathological type 1 FHR gene rearrangements that produce new FHR proteins are involved in genetically driven types of C3G. These rearrangements can lead to the formation of hybrid genes (e.g., CFHR3-1) that disturb the balance of complement regulation by competing with factor H for binding to tissue-bound complement fragments [12,13,14].

The classical clinical picture of the patient with C3G upon admission includes hematuria, proteinuria, and edema, alongside hypertension. Serum C3 levels are decreased in many cases—particularly in DDD (seen in up to 79% of patients) and less frequently in C3GN (about 48%)—which warrants a renal biopsy, which reveals C3 dominant depositions. Clinical presentations can range from isolated microscopic hematuria to nephrotic syndrome or acute nephritic syndrome. Some patients may also develop rapidly progressive glomerulonephritis [12].

Within a decade of being diagnosed, about 30–50% of C3G patients progress to end-stage renal disease (ESRD). When transplantation is provided, there is a high risk of allograft loss. Nonetheless, ongoing clinical trials with new anti-complement therapies have shown promising improvements in the management and outcomes of the disease [12].

### 2.3. Immune-Complex Membranoproliferative Glomerulonephritis

IC-MPGN results from chronic antigenemia due to infections or autoimmunity and is characterized by deposits of immune complexes containing both complement and polyclonal immunoglobulins [15].

Light microscopy shares many features with C3G, yet immunofluorescence differentiates the two by highlighting the presence of C1q, IgA, and IgM [16].

The disease is more frequently encountered in pediatric populations and associated with serologic or genetic evidence of dysregulation of the alternative pathway, with a potential to evolve towards C3G [17].

### 2.4. Membranous Nephropathy

Membranous nephropathy (MN) involves complement activation via anti-phospholipase A2 receptor (PLA2R) antibodies, leading to MAC deposition and podocyte injury. While not yet standard, complement inhibition, particularly targeting C5 and the lectin pathway, holds potential in refractory cases [18,19].

C5b-9, even in sublytic amounts, leads to the activation of various mediators, phospholipases, cyclooxygenases, transcription factors, protein kinases, and cytokines. These signals, in turn, influence the podocyte metabolic pathways, cytoskeletal protein structure and function, nephrin expression and localization, extracellular matrix turnover, and DNA integrity [20,21,22].

There is evidence that podocyte MAC assembly results in the upregulation of the expression of nicotinamide adenine dinucleotide phosphate (NADPH) oxidoreductase and also in its translocation to the cell surface. Reactive oxygen species (ROS) are produced locally and, as a result, lead to lytic processes such as the destruction of membrane proteins, podocyte lipids, and basement membrane components [23,24,25].

Complement-mediated podocyte injury induced by PLA2R antibodies is triggered by the interaction between immunoglobulin G4 (IgG4) and PLA2-R on the podocyte surface. Through binding by MBL, this activates the lectin pathway and leads to the formation of two soluble products, C3a and C5b, and the assembly of the membrane attack complex. C3a and C5a bind to their receptors and, together with the membrane attack complex, cause the degradation of synaptopodin and NEPH1 by cysteine and aspartic proteases [26,27].

### 2.5. IgA Nephropathy and IgA-Associated Vasculitis with Nephritis

Complement activation plays a key role in triggering inflammation and glomerular injury in IgA nephropathy. Over 90% of renal biopsies show glomerular IgA and C3 deposits, while the absence of C3 deposits is associated with a more benign disease course. Complement activation occurs primarily within the kidney, although systemic activation has also been reported [28,29,30,31].

IgAN and IgAVN are associated with mesangial deposition of IgA-containing immune complexes, activating the lectin and alternative pathways. Glomerular deposits of Factor H and properdin—components of the alternative pathway—are commonly observed in IgAN and are linked to disease progression. In addition, up to 40% of IgAN patients exhibit glomerular deposition of C4d, mannose-binding lectin (MBL), and associated serine proteases MASP-1 and MASP-2, components of the lectin pathway, which are associated with poorer prognosis [32,33,34,35].

### 2.6. Systemic Lupus Erythematosus

Systemic lupus erythematosus (SLE) is a complex autoimmune disease in which the complement system plays a dual role: deficiency of early components (C1q, C2, C4) predisposes to disease onset, while overactivation drives inflammation and organ damage, particularly in lupus nephritis and antiphospholipid syndrome [36,37,38].

In individuals with monogenic complement deficiencies—such as mutations in C1q, C1s, C1r, C2, or C4—the inability to clear immune complexes and apoptotic debris leads to uncontrolled immune activation. These patients often present with early-onset, severe SLE, underscoring the essential role of the classical complement pathway in immune homeostasis. As C1q modulates the mitochondrial metabolism of CD8+ T cells, this may explain why C1q has a protective role against SLE. Additionally, lower copy numbers of C4 and C4A are associated with an increased risk of developing lupus, further highlighting the genetic contribution of complement dysregulation [36,37,38].

Conversely, excessive complement activation, particularly via the alternative pathway, exacerbates disease pathology. In lupus nephritis, persistent complement activation leads to glomerular injury, while in antiphospholipid syndrome, complement-mediated endothelial damage contributes to thrombotic events. Low circulating levels of C3 and C4 secondary to complement activation are associated with increased disease activity and are even included in risk scores, with future research directed towards erythrocyte-bound C4d and B cell-bound C4d [39].

From a diagnostic perspective, complement assessment includes measuring C3, C4, and C1q levels, detecting activation markers such as C3a and C5a, and evaluating functional activity via CH50 and AH50 assays. These tests provide crucial insights into disease states and therapeutic responses [39].

Emerging treatments targeting complement regulation offer new therapeutic possibilities. Complement inhibition strategies, such as C5 blockade, are being explored for SLE management, while complement replacement therapies may benefit patients with inherited deficiencies. Understanding the intricate role of complement dysfunction in SLE is critical for advancing diagnostic precision and developing targeted interventions that restore immune balance [39].

### 2.7. Antiphospholipid Antibody Syndrome

The complement system is involved in the pathogenesis of the vascular, obstetric, and catastrophic APS, with evidence of vessel wall depositions and classical pathway activation [40,41,42,43,44].

In quiescent APS, persistently high plasma levels of C5a and sC5b-9 are associated with a higher risk of vascular recurrence. These patients may benefit the most from complement inhibition therapies [45].

In obstetric APS, low preconception C3 and C4 levels may lead to adverse pregnancy outcomes, as are increased Bb and sC5b-9 levels [46,47].

### 2.8. ANCA-Associated Vasculitis

AAV features complement-mediated inflammation via the alternative pathway, with C5a contributing to neutrophil activation. Complement markers (e.g., C3a, C5a) correlate with disease severity. Avacopan (C5a receptor inhibitor) has shown steroid-sparing effects and improved remission rates in clinical trials, including ADVOCATE [48].

In AAV, complement activation occurs mainly through the alternative and terminal pathways. The disease is associated with glomerular staining for FB, properdin, membrane attack complex, and C3d112 and elevated plasma levels of C3a, C5a, and Factor Bb [48].

Glomerulonephritis is generated through the production of C5a and its interaction with its receptor, and given that tumor necrosis factor (TNF) α treated neutrophils with ANCA increased production of C5a, its inhibition and the blockage of the C5a-C5a receptor interaction represents a promising therapeutic perspective [49].

### 2.9. Diabetic Nephropathy

In diabetic nephropathy, complement activation is linked more to the disease progression rather than being a main etiological factor. Several studies have shown that hyperglycemia can cause activation of mannose-binding lectin, and diabetes causes a reduction in CD59 production, events that lead to activation of the complement cascade, producing injuries that cause diabetic nephropathy. In these patients’ renal biopsies, the revelation of focal deposits in the glomeruli and gene expression analysis identified complement activation. However, several other factors, such as age, gender, overweight or obese status, and even infectious processes, influence the complement system activation and response in diabetic nephropathy, along with its potential involvement in adverse events, such as endothelial cell deterioration [6].

Hyperglycemia activates mTORC1 signaling, stimulates STAT1 phosphorylation, and upregulates complement factor B expression, which mediates podocyte injury in diabetic kidney disease [50,51].

Patients suffering from diabetic nephropathy exhibit high levels of C5a, a proinflammatory mediator whose high levels correlate with faster disease progression. In a study conducted by Yiu et al., they showed how C5a signaling inhibition by NOX-D21 also reduced lipid accumulation and TGF-β-driven fibrosis, thus improving tubulointerstitial fibrosis [52].

### 2.10. Focal–Segmental Glomerulosclerosis

Complement involvement in FSGS is limited but present in some patients with histopathological IgM/C3 deposition. Their clinical significance is not yet fully elucidated, yet in comparative studies, patients diagnosed with FSGS with IgM and C3 depositions showed a longer duration of active disease, higher pre-treatment serum creatinine values, and higher rates of segmental and global glomerulosclerosis [53].

An observational retrospective cohort study investigated 70 patients previously diagnosed with primary FSGS proven through renal biopsy and 39 healthy subjects in the control group by measuring plasma and urine levels of C3a, C5a, and soluble C5b-9. The results showed that all marker levels in urine and plasma were increased in the FSGS group compared to the control subjects. Also, plasma and urinary C5b-9 levels were positively related to renal dysfunction, proteinuria, and interstitial fibrosis, and plasmatic C5a level was directly correlated with the degree of segmental glomerulosclerosis [54].

## 3. Emerging Complement System Inhibitors

Given the complexity of the complement system and its contribution to the progression of certain nephrological syndromes, inhibition therapies have emerged which target specific components of the complement cascade and aid in these pathologies [6] (see Table 3).

### 3.1. C3 Inhibition—Pegcetacoplan

Pegcetacoplan, a C3-inhibitor, was investigated in the NOBLE trial, a prospective, phase 2, multicenter, open-label, randomized controlled trial conducted on posttransplant patients with recurrent C3G or IC-MPGN. The patients who received the treatment were investigated after 12 weeks and showed a significant reduction in mean C3G histology activity score and in C3 staining, along with a reduction in urine protein-to-creatinine ratio, stable estimated glomerular filtration rate (eGFR), reduced plasma sC5b-9, and increased serum C3. This trial highlighted the efficacy, safety, and tolerability of pegcetacoplan for patients with posttransplant recurrent C3G and primary IC-MPGN [55].

Similar results were obtained in a study that included patients with four nephropathies: C3G, MN, LN, and IgAN. In the C3G group, pegcetacoplan led to a significant reduction in proteinuria, while serum albumin normalized and eGFR stabilized. Also, serum C3 levels increased, and soluble C5b-9 levels decreased. Reported adverse effects were upper respiratory tract infection, injection site erythema, nausea, and headache. For all four glomerulopathies studied, it had a favorable safety profile [56].

Similar results were observed in pediatric patients diagnosed with C3G, with a significant decrease in proteinuria/creatininuria ratio and erythrocyturia, while patients with impaired kidney function showed an improvement in eGFR, with no adverse effects reported [57].

Improvements in C3G patients under C3 inhibition treatment with pegcetacoplan were also observed in pediatric populations [58].

One study investigated C3 inhibition with pegcetacoplan in both cold agglutinin disease and warm antibody autoimmune hemolytic anemia. Both cohorts of patients had increased hemoglobin levels, reduced hemolysis, and increased functional assessment of chronic illness therapy (FACIT)-fatigue scale scores in the first weeks. These results were maintained long-term [59].

Further studies have shown positive outcomes of pegcetacoplan C3-inhibition for patients with paroxysmal nocturnal hemoglobinuria. This was observed in the PEGASUS trial, where patients experienced an increase in baseline hemoglobin, less dependency on transfusion, lactate-dehydrogenase serum values, reticulocyte count, and improved scores on FACIT-fatigue scales [60,61,62,63,64,65,66].

Other comparative studies showed better results than eculizumab in improving hemoglobin and clinical and hematologic outcomes [67,68,69,70].

Another study examined how venous air embolism, a complication of medical procedures, activates complement and triggers thrombo-inflammation in human blood. Air bubbles significantly increased complement activation, prothrombin fragments, tissue factor mRNA, microparticle tissue factor, β-thromboglobulin, and cytokines. Complement inhibition reduced these effects, with C3 inhibition being the most effective in reducing all mediators [71].

New data from the phase 3 VALIANT trial showed that pegcetacoplan may modify disease progression in patients with C3G and IC-MPGN—particularly those with nephrotic-range proteinuria, a high-risk group for kidney failure. This is the first large-scale randomized trial to test direct C3 inhibition in these complement-driven diseases. In patients with UPCR ≥ 3 g/g, pegcetacoplan led to a 72% reduction in proteinuria at 26 weeks (*p* < 0.0001), with two-thirds achieving ≥50% reduction—none of whom were in the placebo group [72].

Additional benefits included improvements in serum albumin (normalized in 66.7% of treated patients), C3 glomerular staining (improved in 84.6%), and eGFR stability (+16.2 mL/min/1.73 m^2^ vs. placebo). Safety outcomes were favorable, with no excess in adverse events or treatment-related discontinuations. These findings suggest that pegcetacoplan could be a disease-modifying therapy that directly targets complement dysregulation—offering new hope for a population with limited effective treatment options [71].

### 3.2. Factor B Inhibition—Iptacopan

Iptacopan (FABHALTA^®^) is an oral complement Factor B inhibitor, which was approved on 5 December 2023 in the United States for the treatment of paroxysmal nocturnal hemoglobinuria (PNH) in adults. It effectively reduces both terminal complement-mediated intravascular hemolysis and complement 3-mediated extravascular hemolysis by targeting the alternative pathway before complement 5 activation [73].

A phase 3, a double-blind, randomized, placebo-controlled study enrolled adults with biopsies confirming IgA nephropathy within the last 5 years for patients with an eGFR greater than 45 mL and within the last 2 years for patients with an eGFR between 30 and 45 mL if the biopsy showed less than 50% tubulointerstitial fibrosis and urine protein/creatinine ratio greater than 1 despite supportive therapy [74].

Patients were randomized 1:1 to receive 200 mg of Iptacopan twice daily or placebo for 24 months and continued supportive therapy. The primary endpoint was the change in protein/creatinine ratio at 9 months. The primary study population included 222 patients in the Iptacopan group and 221 in the placebo group. After a 9-month period, patients with IgAN who received Iptacopan treatment had a significant reduction in proteinuria compared to the placebo group [74].

Similar results were obtained in another randomized, double-blind, parallel-group adaptive Phase 2 study, conducted on patients with biopsy-confirmed IgAN, eGFR 30 mL/min and above, and urine protein 0.75 g/24 h and over on stable doses of renin-angiotensin system inhibitors [75].

Iptacopan induced statistically significant reductions in average 24 h urine protein-to-creatinine ratio (UPCR) both at three and six months, along with serum levels of Factor Bb and urinary C5b-9. This showed its potential as an alternative pathway inhibitor and a proteinuria reduction agent in IgAN patients [75].

Factor B inhibition is also useful in C3G. In a phase 2, multicenter, open-label, single-arm, nonrandomized study, Iptacopan was prescribed to patients with biopsy-proven, native kidney C3G and to kidney transplant recipients with C3G recurrence. In those with native C3G, UPCR decreased significantly by week 12. In the kidney transplant recipients, reduced C3 deposit scores were observed [76]. The main trials with Iptacopan in C3G are presented in Table 4.

Iptacopan also showed improvements in clinical and biochemical (e.g., hemoglobin) parameters of patients suffering from paroxysmal nocturnal hemoglobinuria and persistent hemolytic anemia [77,78,79,80,81].

### 3.3. C5a Inhibition—Avacopan

Since the alternative pathways are involved in the complement activation in ANCA-associated vasculitis, Avacopan, a selective C5a inhibitor, has been the focus of several trials. In a study by Jayne et al., Avacopan was used instead of glucocorticoids, which adds several side effects. The efficacy of the treatment was determined by achieving a ≥50% reduction in Birmingham Vasculitis Activity Score by week 12 and no worsening in any body system. The control group comprised 23 patients who received a placebo plus prednisone starting at 60 mg daily, along with 22 patients under treatment with Avacopan 30 mg twice daily plus reduced-dose prednisone 20 mg daily, and another 22 who received Avacopan 30 mg twice daily without prednisone. The study observed the clinical response in the Avacopan groups and concluded that in the treatment of ANCA-associated vasculitis, it was an effective alternative to high-dose glucocorticoids [82].

The study by Geetha aimed to demonstrate the safety and efficacy of AVACOPANULI in patients who received induction therapy with Rituximab. The outcome was the remission rate at 26 and 52 weeks. A total of 214 patients received rituximab 375 mg/m^2^ once a week for 4 weeks. Avacopan 30 mg two times a day was administered for 52 weeks in 107 patients, along with glucocorticoids 60 mg/day with dose reduction until discontinuation starting at week 21 [83].

Remission at 26 weeks was observed in 77.6% of the Avacopan group and 75.7% of the prednisone group. Sustained remission at 52 weeks was maintained in 71% of the Avacopan group and 56.1% in the prednisone group. The relapse rate after remission was higher in the prednisone group, 20.2%. An improvement in eGFR at 52 weeks was observed in the Avacopan group by 8.7 mL compared to 6.6 mL in the prednisone group in patients with an eGFR < 30 mL/min/1.73 [83].

Another important trial was ADVOCATE, which included 331 patients diagnosed with ANCA-associated vasculitis and received either Avacopan or prednisone. The results showed a higher increase in eGFR in the Avacopan group by an average of 7.3 mL/min, compared to 4.1 mL/min for the patients who received prednisone [84].

A follow-up analysis only for patients with eGFR under 20 mL/min was conducted, and it indicated an increase of an average of 16.1 mL/min for those who received Avacopan compared to 7.7 mL/min for those who received prednisone, which was also a statistically significant difference [84].

Even in the situation of life-threatening complications of ANCA-associated vasculitis, such as hypoxic pulmonary hemorrhage, Avacopan showed promising results and aided patients in recovering [85].

Such studies have led to Avacopan being recommended by the European Alliance of Associations for Rheumatology (EULAR) to be included in their recommendations for the management of ANCA-associated vasculitis and in the Kidney Disease: Improving Global Outcomes (KDIGO) 2024 Clinical Practice Guideline for the Management of ANCA–Associated Vasculitis [86,87].

Another recent phase 2 randomized, double-blind, placebo-controlled trial assessed the safety and efficacy of Avacopan (30 mg twice daily) in 57 patients with C3 glomerulopathy but found no significant improvement in disease activity or kidney function compared to placebo. Safety profiles were similar between groups, with no new concerns. While the primary endpoint was not met, further studies are needed to evaluate Avacopan’s potential effects on kidney function and disease progression [88].

### 3.4. C5 Inhibition—Eculizumab, Ravulizumab, Crovalimab, Nomacopan

In the case of C5 inhibition, one of the most representative diseases is HUS, which, apart from the kidney, may further affect other organs, especially in pediatric populations. One important therapy which has shown promising results as a C5 inhibitor is eculizumab [89,90].

The humanized monoclonal antibody eculizumab has been used as a C5 inhibitor in patients with paroxysmal nocturnal hemoglobinuria, with promising results and significant improvement. Several patients had a lower response and a poorer prognosis in a study. This was due to the mutation of Arg885, which they all possessed [91,92].

Also, in pediatric study populations suffering from Shiga toxin-related hemolytic uremic syndrome (STEC-HUS) results are mixed. While eculizumab therapy reduced long-term renal sequelae, it did not improve patient outcomes from a nephrological point of view in one study. In other publications, several positive results were noted [93,94,95,96].

In several studies, patients with paroxysmal nocturnal hemoglobinuria had their treatment switched from eculizumab to Ravulizumab, another complement C5 inhibitor. The conclusions were that patients can be switched from eculizumab every two weeks to Ravulizumab every eight weeks without any loss in the positive outcomes and similar safety and tolerability profiles. Despite similar efficacy, patients preferred Ravulizumab due to its administration once every eight weeks, compared to once every two weeks for eculizumab [97,98,99,100,101].

Furthermore, adding Danicopan, a Factor D inhibitor, to the treatment schedule of patients with paroxysmal nocturnal hemoglobinuria and clinically significant extravascular hemolysis alongside eculizumab or Ravulizumab improved hemoglobin concentrations [102].

Another studied C5 inhibitor is Crovalimab, administered every four weeks. It was well-tolerated in most studies on paroxysmal nocturnal hemoglobinuria subjects, and it achieved sustained C5 inhibition [103,104]. It also maintained transfusion avoidance, hemoglobin stabilization, and intravascular hemolysis control. This showed similar results to eculizumab in paroxysmal nocturnal hemoglobinuria but a significant improvement in the FACIT-fatigue scores [105,106,107].

Nomacopan is a C5 inhibitor which, in one case report study, successfully treated a paroxysmal nocturnal hematuria patient, previously non-responsive to eculizumab [108].

In larger studies, it showed good long-term safety and tolerability profiles since it can also be self-administered, along with the therapeutic benefits [67].

Nomacopan was also studied in traumatic hemorrhage patients who had terminal complement activation and emerged as a pro-survival and organ-protective drug, reducing morbidity and mortality, as early post-traumatic C5 inhibition displayed a protective role [109].

### 3.5. Factor D Inhibition—Danicopan, Vemircopan

As mentioned previously, the addition of Danicopan alongside C5 inhibitors in patients with paroxysmal nocturnal hemoglobinuria further improved the outcomes and its benefits were confirmed by other studies. Also, in PNH patients already under C5 inhibitor treatment who continue to experience extravascular hemolysis and require blood transfusions, the addition of Danicopan significantly increased hemoglobin levels, lessened transfusion requirement, and improved FACIT scores [102,107].

The main pathological mechanisms involve controlling intravascular hemolysis and preventing C3-mediated extravascular hemolysis. These effects were confirmed in patients with PNH, as they showed a reduction in intravascular hemolysis, along with a further decrease in mean lactate dehydrogenase levels [110].

Another Factor D inhibitor, Vemircopan, is currently being investigated in the treatment of IgAN and proliferative lupus nephritis [111,112].

### 3.6. MASPs Inhibition—Narsoplimab

For the treatment of thrombotic microangiopathy, the mannan-binding lectin-associated serin protease-2 inhibitors have been studied with promising results. Narsoplimab was studied in a population of patients suffering from hematopoietic stem-cell transplantation-associated thrombotic microangiopathy and showed that it was safe, leading to improvement in TMA markers, i.e., platelet count and lactate dehydrogenase levels, as well as favorable clinical response and overall survival. From a nephrological standpoint, several case reports indicate a resolution of proteinuria [113,114,115].

A summary of the described therapies can be seen in Table 5.

Despite their clinical efficacy, complement inhibitors are associated with significant costs that may limit accessibility, especially in healthcare systems with constrained budgets. Agents such as eculizumab, Ravulizumab, and pegcetacoplan are among the most expensive therapies in nephrology, with annual treatment costs often exceeding several hundred thousand euros per patient. While they have revolutionized the management of complement-mediated kidney diseases, these agents carry significant infection risks, particularly from encapsulated organisms like Neisseria meningitidis, necessitating vaccination and close monitoring. Other safety concerns include infusion reactions, common adverse symptoms (like headache, nausea, hypertension), allergic reactions, and rare laboratory abnormalities such as neutropenia or liver enzyme elevations (see Table 6) [116].

Therefore, the decision to initiate complement inhibitor therapy must balance clinical benefits against financial costs and potential side effects, emphasizing the need for individualized, risk-based therapeutic strategies [5,6].

## 4. Conclusions

Complement inhibition therapies are significantly improving the outcomes in glomerulopathies, especially those with a strong complement involvement in their pathogeny. Studies have shown promising results for C3 inhibition with pegcetacoplan in C3G patients, Factor B inhibition with Iptacopan in IgAN patients, and C5a inhibition with Avacopan in AAV.

Complement inhibitors have revolutionized therapy for complement-driven kidney diseases like C3G, aHUS, and AAV. These agents offer targeted, mechanism-based treatment options with proven efficacy. However, expanding their use to other glomerular diseases requires further research. Identification of disease-specific complement activation profiles will be crucial for precision medicine approaches. Continued investigation into long-term outcomes and cost-effectiveness is essential to fully integrate complement inhibition into nephrology practice.

## Figures and Tables

**Table 1:** Complement regulatory factors and their roles.

Regulatory Factor	Role
Factor I	Degradation of C3b and C4b
Factor H and Factor R	Promotes the dissociation of the C3 convertase of the alternative pathway
C4b-binding protein (C4BP)	Inhibits the classical and lectin pathways and also regulates the alternative pathway by acting as a cofactor for Factor I in C3b degradation
MCP (Membrane Cofactor Protein, CD46)	Binds C3b and C4b and acts as a cofactor for Factor I in both the classical and alternative pathways
DAF (Decay-Accelerating Factor, CD55)	Accelerates the dissociation of C3/C5 convertases in both the classical and alternative pathways
Thrombomodulin (THBD)	Enhances the cofactor activity of Factor H and promotes the inactivation of the anaphylatoxins C3a and C5a
CD59 (Protectin)	Prevents the incorporation of C9 into the C5b-8 complex, thereby inhibiting the formation of the membrane attack complex (MAC)
Vitronectin (Protein S)	Binds to C5b-7 and C5b-8 complexes, preventing C9 polymerization and insertion into the membrane
Clusterin (SP-40)	Inhibits MAC formation by binding to C5b-7 and C5b-8 complexes and preventing their membrane insertion

**Table 2:** The degree of involvement of the complement system in renal diseases.

High Complement Involvement	Intermediate Complement Involvement	Low Complement Involvement
Atypical hemolytic uremic syndrome C3-glomerulopathy Primary immune complex MPGN	ANCA-associated vasculitis IgA nephropathy Systemic lupus erythematosus Antiphospholipid antibody syndrome Membranous nephropathy Secondary TMA Secondary MPGN	Diabetic nephropathy Focal–segmental glomerulosclerosis

**Table 3:** Therapeutic complement inhibitors.

Target	Inhibitors
Factor B	Iptacopan
Factor D	Danicopan, Vemircopan
MASP-2	Narsoplimab
C3, C3b	Pegcetacoplan
C5	Crovalimab, Eculizumab, Nomacopan, Ravulizumab
C5a	Avacopan

**Table 4:** Main trials with Iptacopan in C3G.

Study/Phase	Population	Treatment Duration	Key Outcomes
Phase 2 (Proof-of-concept)	26 patients with native or recurrent C3G	12 weeks	- A 45% reduction in proteinuria (native C3G) - Decreased glomerular C3 deposition scores (recurrent posttransplant C3G)
Phase 2 Extension Study	Same cohort	12 mo	- A 57% reduction in UPCR - Stabilization of eGFR - Increased serum C3 levels (native C3G) - Slight, non-significant proteinuria reduction (transplant cohort) - Stable mean eGFR (transplant) - Significant rise in serum C3 - Improved C3 deposition scores on biopsy
Phase 3 (APPEAR-c3G study)	Patients with C3G	12 mo	- A 35% proteinuria reduction at 6 months, sustained at 12 months - eGFR stabilization - Reduction in C3 deposition vs. placebo
FDA approval granted in March 2025 for the treatment of C3G			

**Table 5:** Clinical evidence summary.

Disease	Effective Inhibitors	Outcomes
C3G	Pegcetacoplan, Iptacopan	↓ Proteinuria, stabilized eGFR
aHUS	Eculizumab, Ravulizumab	Improved renal function, ↓ TMA
AAV	Avacopan	Remission, steroid-sparing, renal protection
IgAN	Iptacopan	↓ Proteinuria
SLE (Lupus Nephritis)	Investigational C5 inhibitors	Ongoing trials
MN	Investigational (C5/lectin pathway inhibitors)	Under investigation
DN	Investigational (C5a, MASP inhibitors)	Adjunctive role under study
FSGS	None established	Research phase

**Table 6:** Adverse events related to complement inhibitor therapy.

Drug	Adverse Event
Pegcetacoplan (C3 inhibitor)	- Infections by encapsulated bacteria (e.g., *Neisseria meningitidis*) - Injection site reactions (erythema, pain, swelling) - Upper respiratory tract infections - Gastrointestinal issues (nausea, diarrhea) - Headache and fatigue - Hypersensitivity reactions (rare) - Artificially prolonged aPTT (can interfere with coagulation tests)
Iptacopan (Factor B inhibitor)	- Infections by encapsulated organisms - Hemolysis (especially in patients with underlying PNH or related disorders) - Hypercholesterolemia - Headache, fatigue - Gastrointestinal symptoms (mild)
Eculizumab and Ravalizumab (C5 inhibitor)	- Meningococcal infections (black-box warning, even after vaccination) - Other infections (e.g., *Haemophilus influenzae*, *Streptococcus pneumoniae*) - Infusion-related reactions (fever, chills, rash, anaphylaxis) - Headache (most common) - Hypertension, nausea, back pain - Neutropenia (rare), elevated liver enzymes
Avacopan (C5a receptor inhibitor)	- Liver function abnormalities (elevated ALT, AST) - Infections (non-specific) - Nasopharyngitis, headache - Hypertension - Nausea, diarrhea - Hypersensitivity (rash, pruritus) - Lower risk of serious infections compared to anti-C5 therapies - Not associated with meningococcal infection risk

## Data Availability

No new data were created or analyzed in this study.

## Abbreviations

The following abbreviations are used in this manuscript:

AAS	antiphospholipid antibody syndrome
AAV	ANCA-associated vasculitis
aHUS	atypical hemolytic–uremic syndrome
ANCA	anti-neutrophil cytoplasmic antibody
C3G	C3-glomerulopathy
DAMP	Damage-associated molecular pattern
DN	diabetic nephropathy
EULAR	European Alliance of Associations for Rheumatology
FACIT	functional assessment of chronic illness therapy
FSGS	focal–segmental glomerulosclerosis
IC-MPGN	immune complex membranoproliferative glomerulonephritis
IgA	immunoglobulin A
IgAN	IgA nephropathy
IgAVN	IgA-associated vasculitis with nephritis
IgG4	Immunoglobulin G4
IgM	Immunoglobulin M
KDIGO	Kidney Disease: Improving Global Outcomes
MAC	Membrane attack complex
MN	membranous nephropathy
MPGN	membranoproliferative glomerulonephritis
NADPH	nicotinamide adenine dinucleotide phosphate
PAMP	Pathogen-associated molecular pattern
PLA2R	Phospholipase A2 receptor
PNH	Paroxysmal nocturnal hemoglobinuria
ROS	Reactive oxygen species
SLE	systemic lupus erythematosus
STEC-HUS	Shiga toxin-related hemolytic uremic syndrome
TCC	Terminal complement complex
TMA	thrombotic microangiopathy
